# The Impact of Cervical Cytology Category Imbalance on Self-Supervised Representation Learning

**DOI:** 10.34133/csbj.0048

**Published:** 2026-04-24

**Authors:** Shijie Liu, Xuben Chang, Jiaxin Bai, Li Chen, Shaoqun Zeng, Tingwei Quan, Xiuli Liucor, Shenghua Cheng

**Affiliations:** ^1^ MoE Key Laboratory for Biomedical Photonics, Wuhan National Laboratory for Optoelectronics, Huazhong University of Science and Technology, Wuhan 430074, Hubei, China.; ^2^ Department of Clinical Laboratory, Tongji Hospital, Tongji Medical College, Huazhong University of Science and Technology, Wuhan 430030, Hubei, China.; ^3^ School of Biomedical Engineering and Guangdong Provincial Key Laboratory of Medical Image Processing, Southern Medical University, Guangzhou 510515, Guangdong, China.

## Abstract

The method of pre-training on extensive unlabeled data, followed by transferring the learned representations to downstream tasks with limited labeled data, has been effective in various fields. However, this paradigm faces the challenge of extreme data imbalance in cervical cytology, with positive cells in whole-slide images constituting approximately 1%. In this paper, we propose a pipeline for investigating the impact of this extreme category imbalance on self-supervised representation learning (SSRL). The pipeline consists of 2 stages: SSRL and downstream tasks. In the SSRL stage, we employ 2 well-established methods, masked autoencoders and the simple framework for contrastive learning, across 9 datasets with varying degrees of imbalance. The pre-trained representations are then transferred to downstream tasks by employing both linear probing and fine-tuning techniques. Additionally, we examine the effect of SSRL on annotation efficiency by varying the quantities of annotation (annotation budget). Our investigation leverages a total of 168,000 image tiles derived from 1,320 whole-slide images obtained from multiple centers. Our findings indicate a noticeable decline in accuracy (Acc) within downstream tasks as data balance shifts from 1:1 to 1:100, with a maximum drop of about 4%. This highlights the substantial impact of data imbalance on SSRL, particularly evident in downstream tasks with lower annotation rates, such as at a 1% budget. Furthermore, the downstream tasks demonstrate the potential to achieve accuracy comparable to those of scenarios with a high annotation budget (50%), even when utilizing a limited annotation budget (5%). The code is available at https://github.com/LGBluesky/ICISSRL.

## Graphical Abstract

**Figure:**
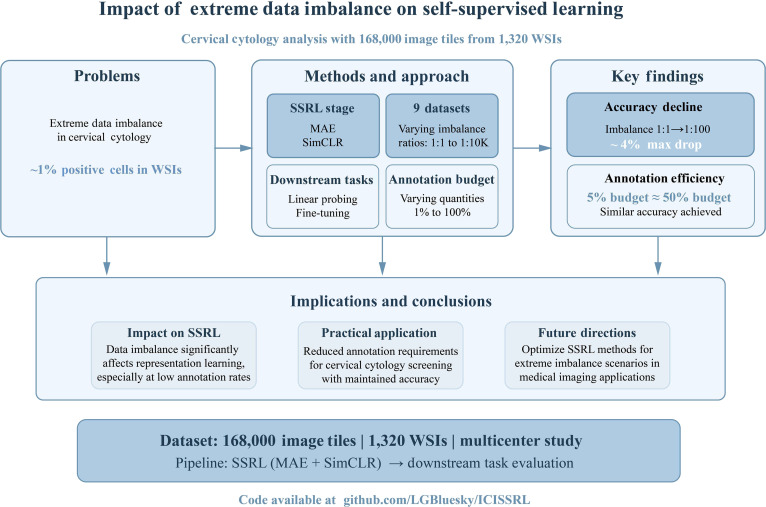

## Introduction

Self-supervised representation learning (SSRL) has achieved promising results on computer vision [1–4], natural language processing [5–7], and speech recognition tasks [8,9]. SSRL harnesses unsupervised techniques to train models through pretext tasks on unlabeled data [10–13]. This enables models to acquire meaningful representations that can subsequently be transferred to a range of supervised downstream tasks. The paradigm that first pre-trains a model through SSRL on a large dataset like ImageNet-1K [14] and in turn fine-tunes the pre-trained weights of the model on the target task has led to impressive results. The success of this paradigm in various fields hinges on the assumption of a similar data distribution between the SSRL and downstream tasks. This similarity ensures the relevance of features extracted during SSRL to specific tasks. With a foundational comprehension of data structures obtained in the SSRL stage, models can rapidly transfer to downstream tasks with minimal fine-tuning using labeled data.

**Fig. 1.**
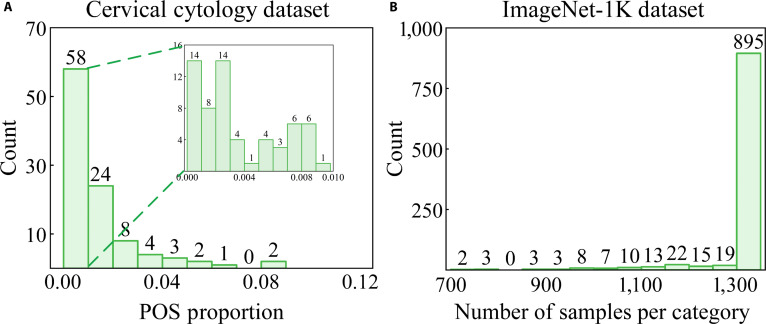
A comparison of category distribution in cervical whole-slide images (WSIs) and the ImageNet-1K [14] dataset. (A) The prevalence of positive cells is comparatively modest, with most WSIs exhibiting a positive cell proportion of less than 1%. The category distribution of cervical cytology exhibits a pronounced imbalance, with a ratio of approximately 1:99 between positive and negative categories. (B) In contrast, in the ImageNet-1K dataset, the allocation of samples among 1,000 categories demonstrates a relatively equitable distribution, with the highest category imbalance approximately reaching only 1:2 (700/1,300). The cervical WSI dataset includes 102 cytology slides (0.243 μm/pixel) collected from multiple centers, scanned with a 3DHISTECH scanner (20×/0.75 numerical aperture [NA]). Cell counts are estimated using nuclear area as a surrogate due to the difficulty of manual counting in WSIs.

Most self-supervised-learning-related work [1,2,15–19] typically operates under data category balance or approximate balanced modes, such as ImageNet-1K [14]. The ImageNet-1K dataset exhibits a maximum category imbalance of 1:2, where the quantity of samples in the smallest category is half as much as the largest category, and notably, 80% of the categories contain an equal number of samples (Fig. 1B). Furthermore, the relevant downstream tasks are executed in a manner that maintains similarity in data distribution with the SSRL stage, employing a data category balanced mode. However, the existing literature [20–22] in SSRL often overlooks a critical issue: the imbalance in data categories during the SSRL stage may result in a disparity between the features learned and the data distribution in downstream tasks.

**Fig. 2.**
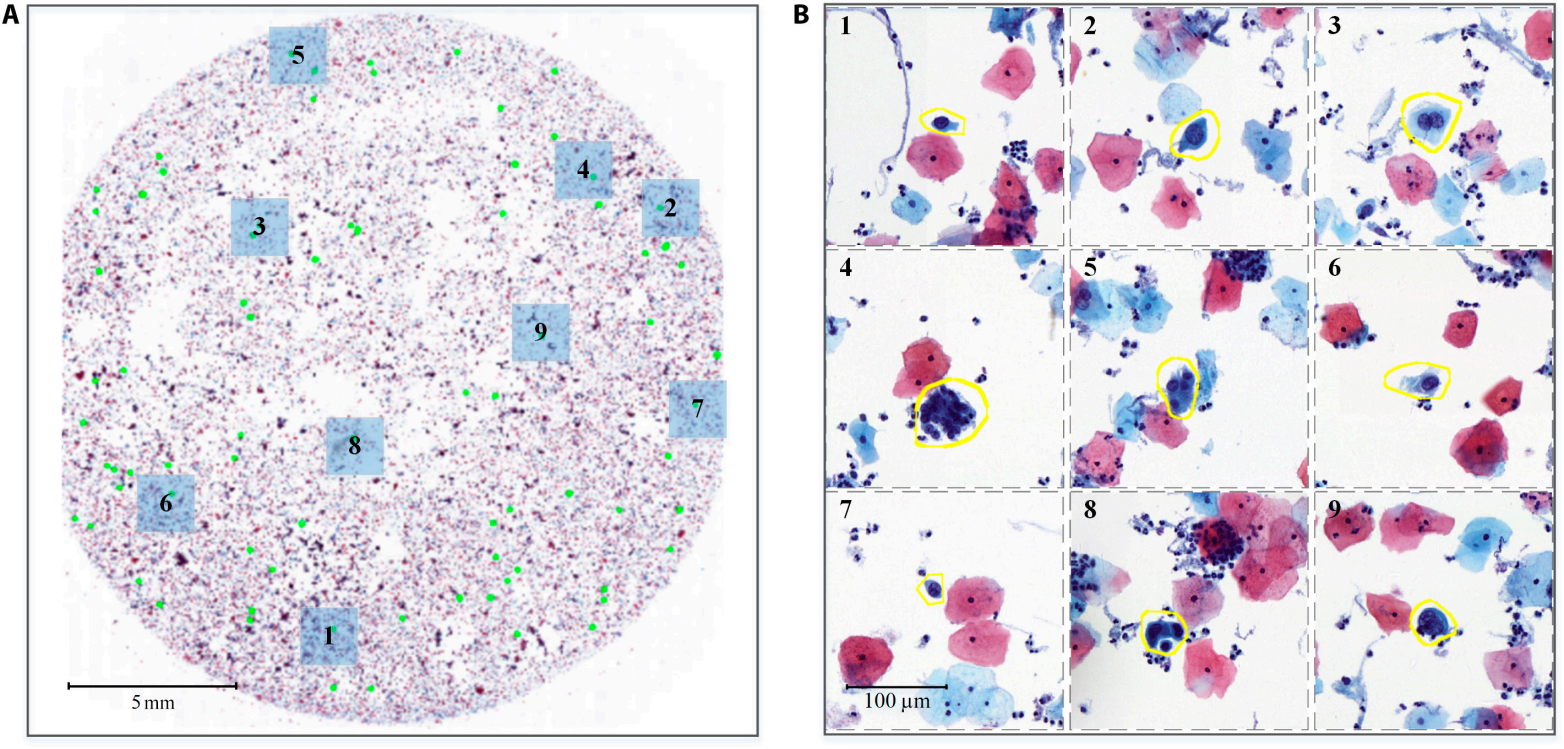
The distribution of positive cells in a whole-slide image. (A) displays a whole-slide image containing 80 positive cells, with an imaging resolution of 0.243 μm/pixel. The proportion of positive cells is merely 0.12%. Green dots present the regions where positive cells are located. (B) provides enlarged views of the 9 positive cell regions from (A), with yellow highlighting indicating the location of positive cells. Each enlarged view is sized at 512 × 512 pixels.

In the field of computational pathology, there is a growing interest in applying SSRL [23–25]. Nonetheless, these works often implement the SSRL paradigm without sufficiently considering the potential impacts of data category imbalance on model training and performance. This issue is particularly salient in the field of cervical cytology, where data characteristics present extremely imbalanced distribution. Indeed, the distribution of positive and negative cells in cervical whole-slide images (WSIs) is extremely unbalanced compared to that in ImageNet-1K [14]. Typically, the proportions of positive cells in cervical WSIs are around 1% (Fig. 1A). Moreover, this estimation is derived from typical lesion slides encountered in clinical settings. In reality, the actual proportion of positive cells in certain cases can be as low as 0.1%, consistent with the proportion depicted in Fig. 2.

During the SSRL stage, the manual endeavor to balance the dataset remains unfeasible, given that the image tiles designated for training are subject to random cropping from WSIs without the incorporation of labeling. Therefore, in the context of this paper, the impact of cervical cytology category imbalance on SSRL is not a direct label-aware effect on the SSRL objective itself. Instead, category imbalance in cervical cytology changes the latent positive-to-negative composition of the unlabeled pre-training data, which introduces sampling bias during pre-training and may lead to biased learning of representations, with a particular deficit in the representation of the positive category. This bias can further extend its influence to downstream tasks, such as linear probing and fine-tuning, especially when dealing with limited annotations. In other words, the impact of cervical cytology category imbalance on SSRL is manifested through its effect on the learned representations and their subsequent transferability.

In this paper, we investigate the impact of category imbalance in cervical cytology on SSRL across 2 stages. Our work utilizes a multicenter dataset derived from 1,320 WSIs, comprising 168,000 image tiles, each with a size of 512 × 512 (0.293 μm/pixel) pixels. This multicenter dataset is divided into 3 parts: 120,000 image tiles for SSRL, 40,000 image tiles for downstream tasks, and an additional 8,000 image tiles for comprehensive evaluation. In the SSRL stage, we establish the positive and negative distributions of cervical cells across multiple ratios. Specifically, we generate 9 sets of data with varying positive and negative cell distributions, ranging from 1:1 to 1:10,000, to simulate the transition of cervical cytology category distribution from balance to extreme imbalance. The aim of this stage is to explore feature representations under varying imbalanced category distributions. Following this, we transfer the pre-trained representations acquired through SSRL to subsequent downstream tasks for further investigating the impact of category imbalance on SSRL. Specifically, we employ linear probing and fine-tuning techniques in downstream tasks. Furthermore, we delve into the influence of SSRL on annotation efficiency by varying annotation budget allocated to these downstream tasks.

The main contributions of this paper are as follows:

• We propose a pipeline to investigate the impact of the category imbalance in cervical cytology on SSRL. To the best of our knowledge, this is the first effort investigating the impact of category imbalance on this SSRL paradigm.
• We investigate the impact of category extreme imbalance on SSRL. Our analysis shows that accuracy (Acc) decreases notably, up to 4%, as the data balance shifts from a 1:1 ratio to a highly imbalanced 1:100. This illustrates the critical impact of data imbalance on SSRL, with the most pronounced effects observed in downstream tasks that have lower rates of annotations, specifically those with only a 1% annotation budget.
• We investigate the impact of SSRL on annotation efficiency in downstream tasks. The results from these downstream tasks demonstrate the feasibility of attaining accuracy levels similar to those in high-annotation-budget scenarios (50%) while operating with a substantially lower annotation budget (5%).

## Related Work

As we focus on deep-learning-based approaches to cervical cell recognition, we omit statements related to traditional rule-based approaches to cervical cell recognition. In the field of cervical cell recognition, most deep-learning-based methods use convolutional neural networks, and transformers have performed well in image classification problems in recent years.

### The paradigm of SSRL and fine-tuning

Generative- and contrastive-learning-based SSRL methods have garnered substantial attention among researchers [1,2]. Generative SSRL methods aim to reconstruct masked portions of an image using the unmasked regions. Early efforts attempted to utilize convolutional neural networks for reconstructing masked areas in images; however, these methods yielded moderate success [26]. Recently, with the emergence of vision transformers [27] in the field of computer vision, there is a growing trend in utilizing transformers as the fundamental building block for network architectures. Notably, recent work such as BERT pre-training of image transformers [19] and masked autoencoders (MAEs) [1] demonstrated state-of-the-art performance across various benchmark datasets of natural images.

The assumption of contrastive-learning-based SSRL methods is that data transformations applied to images do not alter their semantic information [28,29]. The core idea of such methods is to construct positive and negative pairs through data transformations and then to optimize the model by minimizing the distance between positive pairs while pushing apart the negative samples in the latent space. The simple framework for contrastive learning (SimCLR) [2] stands as a substantial representative of contrastive-learning-based SSRL methods, achieving outstanding performance on large-scale datasets like ImageNet with substantially less labeled data, approximately 100 times fewer annotations compared to those in supervised methods. However, a limitation of SimCLR is its relatively high dependency on a large batch size, which somewhat restricts its application in resource-constrained environments and increases the demand for hardware resources. To overcome this limitation, MoCo [16] introduced a method called momentum contrast, which extends the number of negative samples by using a momentum-encoded queue. This innovation reduces the reliance on a large batch size, allowing MoCo to achieve excellent performance even in resource-constrained settings. Recent work, such as BYOL [18] and DINO [17], further mitigated the dependence on large batch sizes by employing different strategies to increase the number of negative samples.

In this work, we employ 2 SSRL methods, MAE and SimCLR, to investigate the impact of category imbalance in cervical cytology on SSRL. These methods are chosen for their wide application across various fields and their ease of deployment.

### The application of SSRL in pathology

SSRL methods are gradually gaining widespread application in the field of pathology. Li et al. [24] successfully applied SimCLR [2] to multiple pathology image datasets, improving the accuracy of classification tasks on these datasets. Additionally, Saillard et al. [23] employed MoCo V2 [30] for SSRL on The Cancer Genome Atlas dataset (https://www.cancer.gov/tcga), substantially enhancing the accuracy of microsatellite instability detection tasks. Stegmuller et al. [25] also incorporated DINO [17] into feature extraction for cervical cell images, effectively reducing the challenges associated with human papillomavirus screening in resource-limited and data-scarce environments. However, these methods associated with histopathology overlooked the impact of category imbalance on model performance. This oversight arises from extensive lesions within histopathology images, leading to a comparatively less pronounced class imbalance between positive and negative categories across the whole slide, as opposed to the more acute scenario observed within the cervical cytology domain. It is particularly noteworthy that in cervical cytology, there is an extreme imbalance in the distribution of positive and negative cells, presenting a challenge for SSRL.

## Methods

**Fig. 3.**
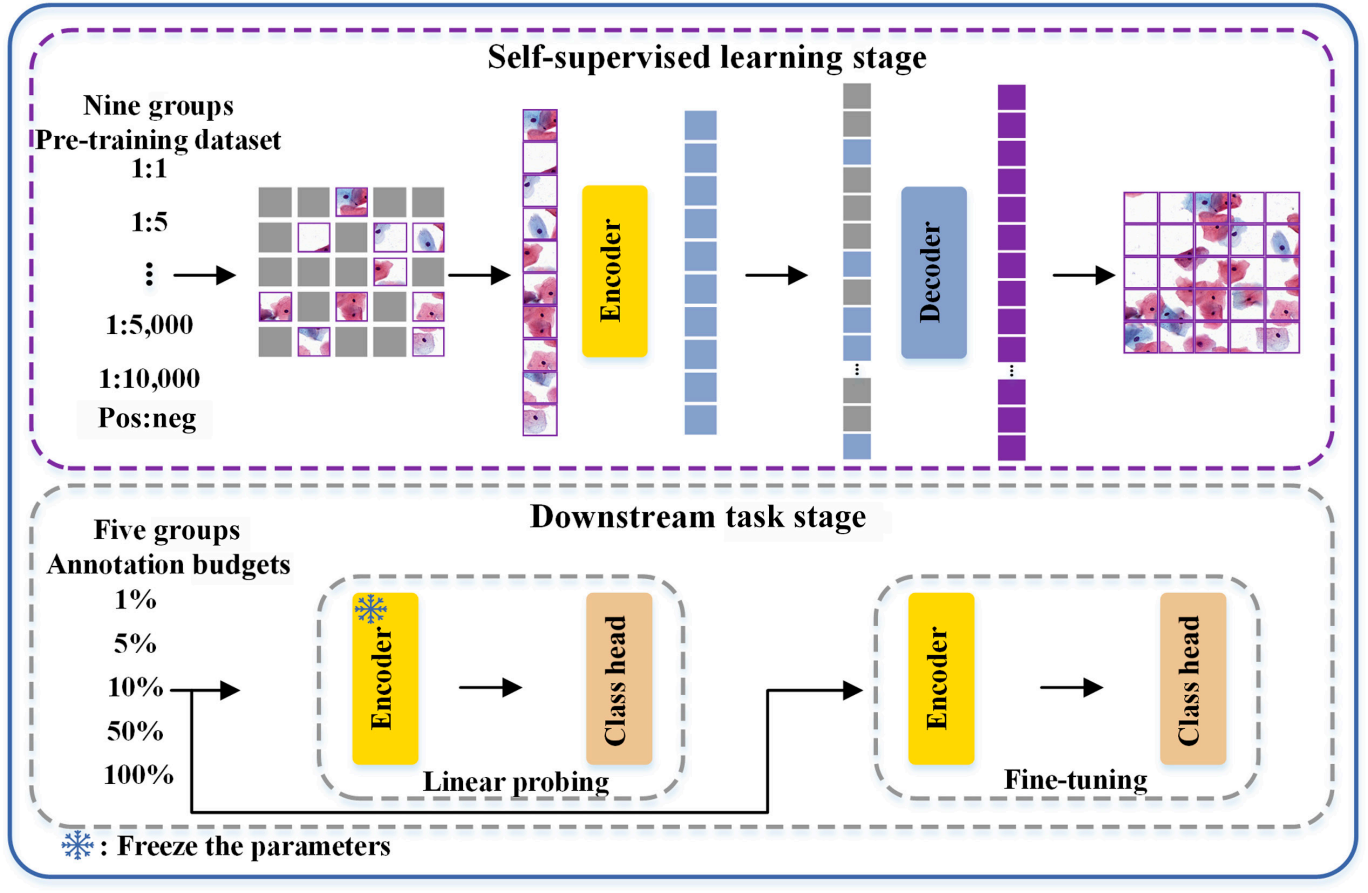
The pipeline for investigating the impact of extreme category imbalance in cervical cytology on self-supervised representation learning (SSRL) and subsequent downstream tasks.

The principal aim of this work is to investigate the impact of extreme category imbalance in cervical cytology on SSRL. More specifically, under the title-level question of how cervical cytology category imbalance affects SSRL, this work focuses on the mechanism by which category imbalance changes the latent positive-to-negative composition of the unlabeled pre-training data and thus affects SSRL through sampling bias during pre-training. Figure 3 illustrates the pipeline of this work, which consists of 2 stages: (a) the SSRL stage and (b) the downstream task stage. During the SSRL stage, we investigate feature representations under varying imbalanced category distributions. The pre-trained representations acquired at this stage represent the various distribution of category distributions. Subsequently, these pre-trained representations are transferred to downstream tasks to further investigate the impact of category imbalance within cervical cytology on SSRL. Furthermore, we delve into the influence of SSRL on annotation efficiency by allocating various annotation budgets in these downstream tasks.

### Self-supervised representation learning

During the SSRL stage, it is essential to construct datasets that mimic the category imbalance in cervical cytology. Specifically, we generate 9 datasets with diverse distributions of cervical cytology categories. It should be noted that these positive-to-negative ratios are used only to control the semantic composition of the unlabeled pre-training data. The category labels are required for offline dataset construction, but they are not exposed to the self-supervised models during optimization. Therefore, the impact of cervical cytology category imbalance on SSRL in this work is studied as an implicit effect induced by sampling bias in unlabeled pre-training data, rather than as a direct label-aware modification of the SSRL objective. Subsequently, we employ 2 well-established SSRL methods, MAE and SimCLR, to examine their individual impacts.

#### Generative SSRL

Generative SSRL methods strive to reconstruct masked regions of an image using the unmasked regions. In our implementation, we employ MAE [1] as one generative SSRL method. Specifically, it utilizes the reconstruction loss $L_{\text{recon}}$ to reconstruct the masked regions $X_{M}$ from the image to the unmasked regions ${\tilde{X}}_{M}$. Since MAE learns by reconstructing visual content rather than using category labels, the effect of cervical cytology category imbalance on MAE is reflected through the skewed composition of the unlabeled pre-training data and the resulting representation bias. The model parameters are obtained through the following optimization:

$$\theta^{*}=\underset{\theta }{\text{arg}\;\text{min}}\left(L_{\text{recon}}\left(f_{\theta }\left(\cdot \right),\;\left\{\left.\left(x,\;y\right)\right|x={\tilde{X}}_{M},\;y=X_{M}\right\}\right)\right),$$ (1)

where $f_{\theta }\left(\cdot \right)$ represents the MAE network and θ represents the parameters of the network.

#### Contrastive-learning-based SSRL

Contrastive-learning-based SSRL is another widely applied SSRL method [2,31,32]. Its core concept revolves around contrastive learning to acquire feature representations. In our implementation, we employ SimCLR [2] as one contrastive SSRL method. Specifically, we compare different transformations $T_{1}$, $T_{2}$ from the same image X in the latent space by the contrastive loss $L_{\mathrm{con}}$. Since SimCLR organizes the embedding space through instance comparisons rather than category supervision, the effect of cervical cytology category imbalance on SimCLR is also indirect; i.e., it is manifested through the skewed semantic distribution of the unlabeled pre-training data and its influence on learned representations. The model parameters are obtained through the following optimization:

$$\theta^{*}=\underset{\theta }{\text{arg}\;\text{min}}\left(L_{\mathrm{con}}\left(f_{\theta }\left(\cdot \right),\;\left\{\left.\left(x,y\right)\right|x=X_{T_{1}},\;y=f_{\theta }\left(X_{T_{2}}\right)\right\}\right)\right),$$ (2)

where $f_{\theta }\left(\cdot \right)$ represents the SimCLR network and θ represents the parameters of the network.

### Downstream tasks

The downstream tasks further refines the pre-trained representations obtained from the SSRL stage by introducing a limited annotation budget. This process is tailored specifically for a binary classification framework, aiming to enhance the sensitivity of these representations to distinct cervical cytology categories. This enhancement is primarily achieved through 2 techniques: linear probing and fine-tuning. Specifically, linear probing involves freezing the network’s feature extraction layer/module ${f_{\theta }}^{*}$ and optimizing only the classification head $g\left({f_{\theta }}^{*}\left(\cdot \right);\phi \right)$.

In this work, the downstream datasets are kept balanced with a positive-to-negative ratio of 1:1 under all annotation budgets. Therefore, the downstream stage does not introduce additional category imbalance. Under this setting, the observed performance differences mainly reflect the transferability of the pre-trained representations learned from different unlabeled pre-training distributions, namely, the effect of pre-training-induced representation bias.

The model parameters in linear probing are obtained through the following optimization:

$$\phi^{*}=\underset{\phi }{\text{arg}\;\text{min}}\left(L_{\mathrm{ce}}\left(g\left({f_{\theta }}^{*}\left(\cdot \right);\phi \right),\;\left\{\left.\left(x,\;y\right)\right|x\in X,\;y\in Y\right\}\right)\right),$$ (3)

where ϕ represents the parameters of the appended classification head $g\left(\cdot \right)$; the label Y signifies the target classification, with 0 denoting the negative category and 1 denoting the positive category; and $L_{\mathrm{ce}}$ represents the cross-entropy loss function. On the other hand, fine-tuning is not constrained by the network’s structure and can optimize any layers of the network. The model parameters in fine-tuning are obtained through the following optimization:

$$\left[\theta^{*},\phi^{*}\right]=\underset{\left[\theta ,\phi \right]}{\text{arg}\;\text{min}}\left(L_{\mathrm{ce}}\left(g\left(f_{\theta }\left(\cdot \right);\phi \right),\left\{\left.\left(x,y\right)\right|x\in X,y\in Y\right\}\right)\right).$$ (4)

For clarity, the classification head for linear probing inherits from the MAE network and is composed of batch normalization and a linear layer. Fine-tuning is performed by optimizing the entire network.

### Evaluation protocols

This study primarily evaluates model performance using accuracy (Acc), specificity (SP), sensitivity (SE), precision (PR), F1-score (F1), and area under the curve (AUC). In addition, t-distributed stochastic neighbor embedding (t-SNE) [33] is employed as an auxiliary tool for representation analysis rather than as a quantitative evaluation metric. Specifically, t-SNE is used to visualize the high-dimensional image representations in the embedding space after dimensionality reduction, so as to investigate the effectiveness of SSRL and the influence of category imbalance in cervical cytology on the learned representations. Here, this influence refers to the effect of cervical cytology category imbalance on the representations learned during self-supervised pre-training through sampling bias in unlabeled data. The quantitative metrics for downstream tasks are defined as follows:

Acc=TP+TNTP+TN+FP+FN,SP=TNTN+FP,SE=TPTP+FN,PR=TPTP+FP,F1=2TP2TP+FP+FN,AUC=∫01TPRxdFPRx. (5)

## Experiments

This section provides a detailed exposition of experimental data, model parameters, and model evaluations.

### Dataset

#### Data collection

In our experiments, we gathered a dataset of 1,320 WSIs from the Maternal and Child Health Hospital of Hubei Province, affiliated with Tongji Medical College, Huazhong University of Science and Technology. This collection was uniquely sourced from multiple centers within the hospital, representing numerous patient cohorts. These images were collected using sophisticated medical imaging equipment, yielding diverse resolutions: 3DHISTECH at 0.243 μm/pixel (under 20× magnification), the Wuhan National Laboratory for Optoelectronics’s custom-designed imaging system at 0.293 μm/pixel (under 20× magnification), and Shenzhen Shengqiang Technology at 0.180 μm/pixel (under 40× magnification).

#### Data preprocessing

Given that these 1,320 WSIs are sourced from multiple centers, we initially standardize the resolution of 168,000 image tiles derived from these slides to mitigate the potential effects of resolution variability on experimental accuracy, interpolating each image tiles to 0.293 μm/pixel. To avoid potential data leakage, the dataset was first partitioned at the WSI level before tile extraction. Since each WSI corresponds to a unique patient in our dataset, this strategy also guarantees patient-level independence. After the WSI-level split was finalized, tiles were generated independently within each subset, ensuring that no tiles from the same WSI (or patient) appear across different datasets. Subsequently, these image tiles are uniformly cropped to 512 × 512 pixels, conforming to the input specifications of the models utilized in this work. The whole 168,000 image tiles are divided into 3 parts: 120,000 image tiles for SSRL, 40,000 image tiles for downstream tasks, and an additional 8,000 image tiles for comprehensive evaluation, as detailed in Table 1. Furthermore, we randomly select 1,000 positive and 1,000 negative image tiles from the comprehensive evaluation set for feature representations.

**Table 1.** The overview of data division

SSRL	DT	CE	Size	Resolution	WSIs
120,000	40,000	8,000	512 × 512	0.293 μm/pixel	1,320

SSRL, self-supervised representation learning; DT, downstream tasks, CE, comprehensive evaluation; WSIs, whole-slide images

For SSRL, we establish 9 data sampling schemes with positive-to-negative ratios ranging from 1:1 to 1:10,000. These schemes involve sampling from the pool of these 120,000 images tiles, resulting in each experimental dataset containing 80,000 images tiles for training. For the downstream tasks, we divide 5 distinct groups from the pool of 40,000 image tiles, with dataset sizes of 40,000 (100%), 20,000 (50%), 4,000 (10%), 2,000 (5%), and 400 (1%), as detailed in Table 2. Subsequently, we perform 5 random samplings for each group to generate 5 subgroups of data. The data within each subgroup are divided into training and validation sets in a 4:1 ratio, ensuring that the ratio of positive to negative categories remained 1:1 in each set. The evaluation on these downstream tasks is performed on the comprehensive evaluation set, as detailed in Table 1.

**Table 2.** The data division for downstream tasks

Data ID	Number	Annotation budget	Train	Val
1	40,000	100%	32,000	8,000
2	20,000	50%	16,000	4,000
3	4,000	10%	3,200	800
4	2,000	5%	1,600	400
5	400	1%	320	80

### Experimental results

#### Experimental setup

The experimental parameter settings consist of 2 stages: the SSRL stage and the downstream task stage.

During the SSRL stage, we conduct experiments with 2 well-established architectures, ResNet-50 [34] and ViT-B/16 [27], as backbones for MAE and SimCLR, respectively. During the MAE-related process, we set the mask ratio to 0.5 and employ the recommended hyperparameters available on the official repository (https://github.com/facebookresearch/mae). Specifically, we utilize the AdamW [35] optimizer with an initial learning rate of 2.25e−4 and employ a learning rate update strategy that includes warming up and cosine annealing decay. Additionally, we select a batch size of 192 per graphics processing unit (GPU) to fill the available GPU memory, corresponding to a global batch size of 384 under the 2-GPU distributed data parallel training setup. In the SimCLR-related experiments, most parameters remained similar to those of MAE. However, due to the benefits observed in the SimCLR experiments with larger batch sizes, we opt for a higher batch size of 256 per GPU, corresponding to a global batch size of 512, and accordingly adjust the learning rate to 3e−3. The main parameter settings in the SSRL stage are summarized in Table 3, while the detailed training parameters are provided in Tables S11 and S12.

**Table 3.** The parameter settings of SSRL

Model	Backbone	Mask ratio	Lr	Lr_updated	Optimizer	Warm	Epoch	Batch
MAE	ViT-B/16	0.5	2.25e−4	Cosine annealing	AdamW	10	200	384
SimCLR	ResNet-50	-	3e−3	Cosine annealing	AdamW	10	200	512

SSRL, self-supervised representation learning; MAE, masked autoencoder; SimCLR, simple framework for contrastive learning

During the downstream task stage, we employ 2 different optimizers, including SGD [36] and AdamW [35], for linear probing and fine-tuning, respectively. We follow the learning rate setting strategy from MAE [1], which is given by

lr=batch×blr/256, (6)

where blr serves as the base learning rate, which increases with the growth of the batch size. Considering GPU memory capacity and the size of the datasets used in downstream tasks, we set the base learning rate blr to 1.5e−3 for linear probing and 5e−4 for fine-tuning, following the official repository 4.2.1 and adjust the batch size accordingly for various annotation budgets (100%, 50%, 10%, 5%, and 1%), resulting in batch sizes of 384, 256, 64, 64, and 32, respectively. Similarly, in the SimCLR-related downstream tasks, only the batch for the 100% annotation budget differed from the aforementioned settings, with the value set to 512. Additionally, we continue to employ a combined strategy of cosine annealing and warming up for updating the learning rate, with warm-up epochs set to 5. The main settings for the other parameters are summarized in Table 4, whereas the detailed parameter configurations are presented in Tables S13 to S16.

**Table 4.** The parameter settings of downstream tasks

Mode	Blr	Lr_updated	Optimizer	Warm	Epochs	Batch
Linear probing	1.5e−3	Cosine annealing	SGD	5	100	384/512 256 64 64 32
Fine-tuning	5e−4	Cosine annealing	AdamW	5	50	384/512 256 64 64 32

All of the experiments are completed in PyTorch 2.0 with distributed data parallel mode and Ubuntu 20.04 equipped with an Intel i7-6850K central processing unit, 3.60 GHz, 64-GB random-access memory, and 2 NVIDIA RTX 3090 GPUs.

#### Transferring pre-trained representations to downstream tasks

In our implementation, we transfer the pre-trained representations obtained from the SSRL stage to the downstream tasks. During the evaluation process, we assess the effectiveness of SSRL by analyzing the performance of downstream task models. Specifically, we conduct comparisons among models trained with diverse initial weights originating from 2 sources. One set is pre-trained on cervical cytology images, specifically tailored for our work, while the other is inherited from self-supervised weights pre-trained on the ImageNet-1K [14]. In our implementation, we select the pre-trained weights aligned with a 1:1 distribution of cervical cell categories, as the ImageNet-1K inherently maintains an approximate category balance (Fig. 1B). Additionally, for downstream tasks, experimental data with various annotation budgets from 1% to 100% are obtained through 5 rounds of random sampling.

**Fig. 4.**
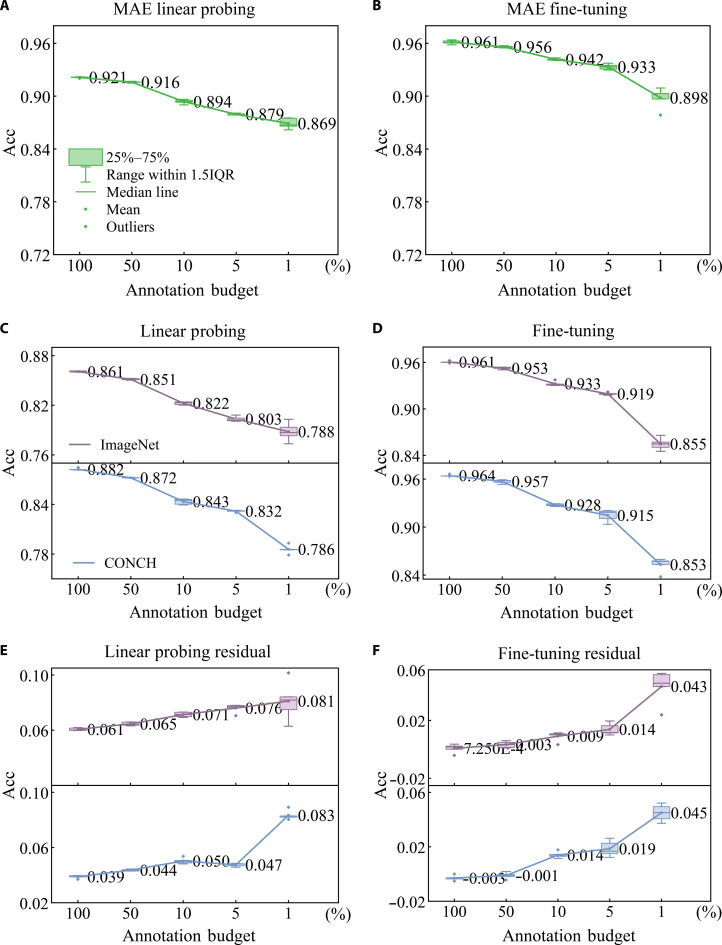
Evaluation of downstream tasks in the masked autoencoder (MAE) framework under balanced category distribution in cervical cytology. (A) illustrates the performance of linear probing, and (B) demonstrates the performance of fine-tuning, both initialized with the weights pre-trained on cervical cytology. (C) and (D), on the other hand, evaluate linear probing and fine-tuning initialized with weights pre-trained on ImageNet-1K [14] and CONCH [21], where ImageNet-1K is shown in purple and CONCH is shown in blue. In the boxes, the *x*-axis represents varying quantities of annotation, where 100% annotation budget corresponds to 40,000 annotations, as shown in Table 2. (E) presents the residual in accuracy (ACC) between (A) and the ImageNet-1K results in (C) (purple), as well as between (A) and the CONCH results in (C) (blue), while (F) presents the corresponding residuals between (B) and the ImageNet-1K results in (D) (purple) and between (B) and the CONCH results in (D) (blue).

**Fig. 5.**
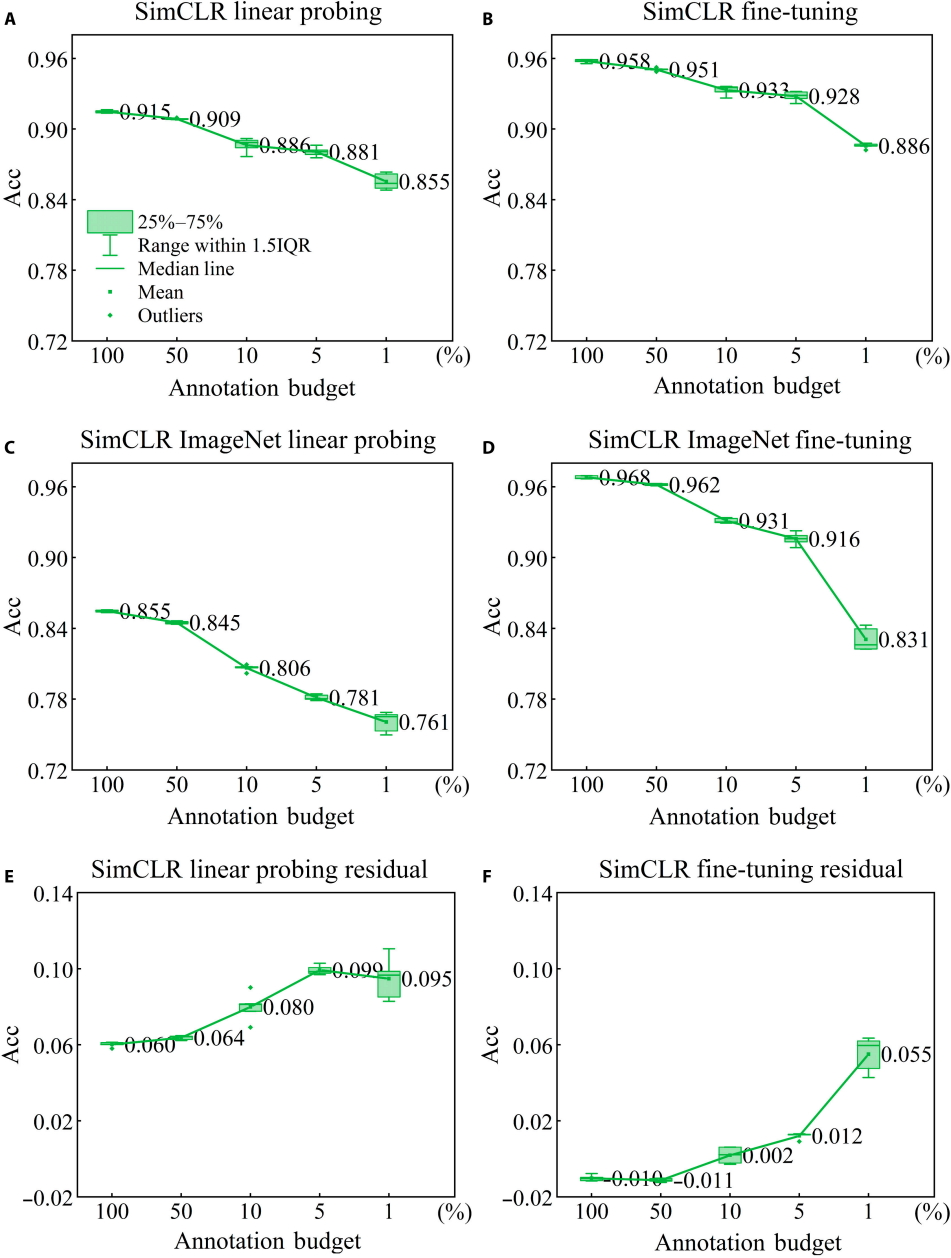
Evaluation of downstream tasks in the simple framework for contrastive learning (SimCLR) framework under balanced category distribution in cervical cytology. (A) illustrates the performance of linear probing, and (B) demonstrates the performance of fine-tuning, both initialized with the weights pre-trained on cervical cytology. (C) and (D), on the other hand, employed the weights pre-trained on ImageNet-1K for linear probing and fine-tuning evaluations, respectively. (E) presents the residual between (A) and (C), while (F) presents the residual between (B) and (D).

We present the performance of downstream tasks using box plots, as shown in Figs. 4 and 5. Since linear probing freezes the network’s feature extraction module, it provides the most reflective assessment of the quality of upstream SSRL.

Figure 4 presents the performance of relevant downstream tasks under the MAE framework. In our comparisons, CONCH, shown in blue, is included as a pathology-domain pre-trained baseline, while ImageNet-1K, shown in purple, serves as a more generic and comparatively weaker baseline. Under the linear probing setting, cervical-cytology-pre-trained weights consistently outperform both baselines, and the advantage becomes increasingly pronounced as the annotation budget decreases. Compared with CONCH, MAE yields superior performance across multiple annotation budgets, particularly in the low annotation budget. Specifically at the 1% annotation budget, for instance, MAE achieves an Acc of 0.869 (Fig. 4A), exceeding CONCH’s Acc of 0.786 (Fig. 4C) by 8.3% (Fig. 4E). Relative to ImageNet-1K, the average Acc gain reaches 7%, increasing from 6% to 8% as the annotation budget drops (Fig. 4E), which further supports the effectiveness of SSRL. Under the fine-tuning setting, the differences in performance between distinct initial weights are relatively small at higher annotation budgets (100% or 50%). As the annotation budget decreases from 10% to 1%, the reliance on the initialization model’s weights in downstream task substantially increases. Compared with both baselines, MAE continues to show an advantage, especially under low annotation budgets. Specifically at the 1% annotation budget, MAE attains an ACC of 0.898 (Fig. 4B), surpassing CONCH by 4.5% and ImageNet by 4.3% (Fig. 4F).

Figure 5 presents the performance of downstream tasks under the SimCLR framework. Similar to Fig. 4, we observe a clear advantage in utilizing the initial weights pre-trained on cervical cytology images (Fig. 5A) in the linear probing task compared to the initial weights pre-trained on ImageNet-1K (Fig. 5B). Additionally, we notice a close correlation between the model’s reliance on annotation budget for weight initialization and performance enhancement. When the annotation budget is as low as 5% or 1%, the model’s performance improves by over 9% (Fig. 5E). However, in fine-tuning tasks, we observe a less pronounced relative enhancement in model performance, with only a 5% increase observed when the annotation budget is set at 1% (Fig. 5F). This phenomenon can be attributed to the fact that fine-tuning task involves the optimization of all model parameters, and as the dataset size increases, the model undergoes more optimization iterations, resulting in diminished performance differences. Both findings indicate that as the annotation budget decreases, the model’s reliance on initial weights becomes more pronounced, leading to a more substantial performance boost.

**Fig. 6.**
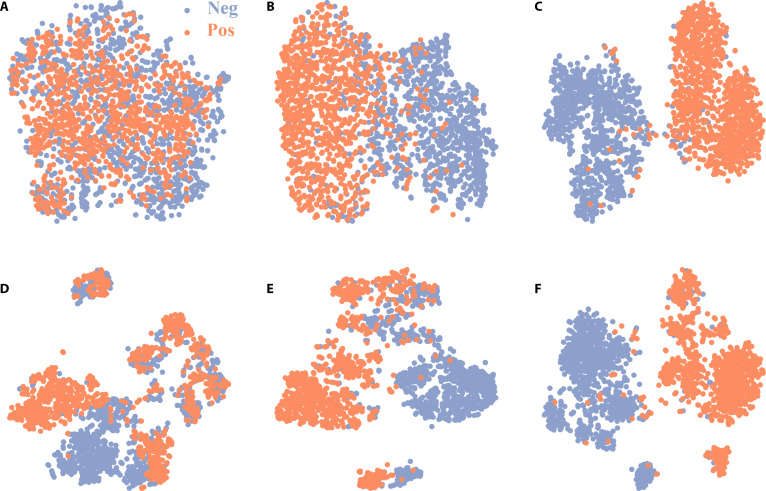
t-distributed stochastic neighbor embedding (t-SNE) projection of learned features under the masked autoencoder (MAE) framework. We reduce the last 768-dimensional features from ViT-B/16 [27] to 2-dimensional (2D) feature space using t-SNE on the selected 2,000 image tiles with a 1:1 ratio of positive to negative samples. (A) Initial stage with the weights pre-trained on ImageNet-1K. (B and C) Fine-tuning based on (A) with 1% and 100% annotation budgets, respectively. (D) Initial stage with the pre-trained weights from cervical cytology aligned to a 1:1 distribution of categories. (E and F) Fine-tuning based on (D) with 1% and 100% annotation budgets, respectively.

Additionally, we employ t-SNE for visualizing image representations before and after fine-tuning, for further investigating the effectiveness of SSRL on cervical cytology images. Specifically, within the framework of MAE, we compared the visualizations of 2 distinct pre-trained representations as well as their corresponding fine-tuned representations in downstream tasks (Fig. 6). In Fig. 6A, we depict the feature representations obtained using the pre-trained weights directly from the ImageNet-1K dataset [14], while Fig. 6D showcases the feature representations acquired from the pre-trained weights specifically tailored for cervical cytology. Compared to the former, the latter exhibits improved separability between positive and negative cells in the 2-dimensional (2D) feature space and a preliminary indication of clustered category aggregation, confirming the effectiveness of SSRL on cervical cytology. Furthermore, we fine-tune the models above with different annotation budgets, where Fig. 6B and E employ a 1% annotation budget, while Fig. 6C and F utilize the full 100%. These fine-tuning operations substantially reduce the confusion between positive and negative cells. Although both methods fine-tune using cervical cytology data, the approach employing SSRL directly on these data (Fig. 6E) demonstrates a substantial advantage, especially when fine-tuned with only a 1% annotation budget, over the method that fine-tunes using the weights pre-trained on ImageNet-1K without prior adaptation to cervical cytology images (Fig. 6B). The reason for this is that with a small annotation budget (1%) for fine-tuning, the model’s performance becomes more dependent on the initial weights, thereby underscoring the value of employing SSRL on cervical cytology data. In contrast to the latter, which exhibits an approximate clustering in the 2D feature space, the former demonstrates a more refined clustering, characterized by distinct clusters. However, when fine-tuning is conducted with higher annotation budgets, the model increasingly learn from the abundant annotations, thereby reducing its dependence on initial weights. This decreased reliance on initial weights explains the lack of substantial performance improvement in the comparison between Fig. 6C and F.

Furthermore, we quantitatively analyzed the t-SNE visualizations in Fig. 6 by assessing feature separability with a linear support vector machine trained in the 768-dimensional embedding space produced by the MAE using 5-fold stratified cross-validation. The linear support vector machine results (Table S17 and Fig. S1) show that a notable improvement occurs during the pre-training stage: MAE-pre-trained embeddings achieve an average accuracy of 0.89, compared with 0.82 for ImageNet-initialized embeddings (*P* = 0.005). Under a 1% annotation budget, MAE embeddings reach an average accuracy of 0.90 versus 0.86 for ImageNet (*P* = 0.014). Across all evaluated metrics, including accuracy, specificity, sensitivity, precision, F1-score, and AUC, MAE consistently outperforms ImageNet, indicating stronger linear separability. These results are consistent with the t-SNE patterns shown in Fig. 6, further supporting that the 2D visualizations faithfully reflect the intrinsic separability of the embeddings.

In summary, we find that transferring pre-trained representations from cervical cytology images to subsequent downstream tasks substantially enhances task performance, particularly in scenarios with limited annotation by employing 2 distinct initial weights in downstream tasks. This further validates the effectiveness of conducting SSRL on cervical cytology images.

#### Quantifying the impact of category imbalance on self-supervised representations in downstream tasks

After validating the effectiveness of SSRL, we delve into the impact of the category imbalance in cervical cytology on self-supervised representations in downstream tasks. Recognizing that this imbalance does not directly affect these tasks, we adopted a novel strategy. We consider the pre-trained representations, obtained during the SSRL stage under various category distributions of cervical cytology, as an alternative representation of category distribution. These pre-trained weights are then utilized as initial weights for the models in downstream tasks. We conduct experiments on downstream tasks separately in 2 different SSRL frameworks, namely, MAE and SimCLR. The ratio of positive to negative samples in cervical cytology images is employed to quantify the degree of category imbalance.

**Fig. 7.**
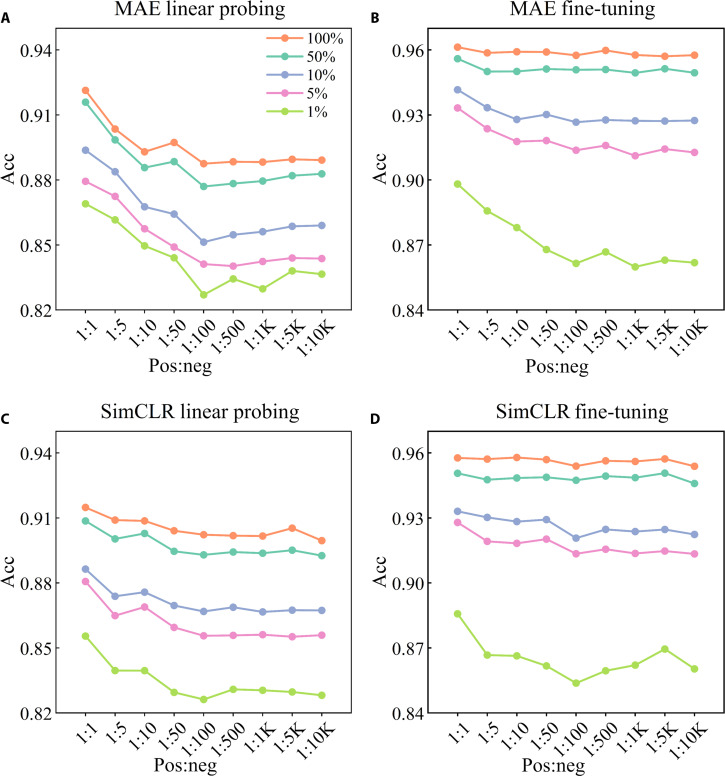
Comprehensive evaluation of downstream tasks. This assessment illustrates the impact of the imbalance in cervical cytology category on the performance of downstream task. (A) and (B) respectively present the evaluation results of linear probing and fine-tuning under the masked autoencoder (MAE) framework, while (C) and (D) respectively showcase the results of linear probing and fine-tuning under the simple framework for contrastive learning (SimCLR) framework. The *x*-axis represents the distribution of cervical cytology category, with “K” denoting 1,000. The legend represents the 5 distinct annotation budgets in downstream tasks, with 100% corresponding to 40,000 annotations.

Figure 7 presents the comprehensive evaluation of downstream tasks for 5 distinct annotation budgets, under 2 different SSRL frameworks, corresponding to 9 pre-trained weights, each associated with a specific cervical cytology data distribution. Since fine-tuning involves optimizing all parameters of the models, the evaluation results (Acc curves) of fine-tuning (Fig. 7B and D) are comparatively smoother compared to those of linear probing (Fig. 7A and C, especially when there is an adequate annotation budget (100% or 50%). The figure delineates the mean Acc values derived from 5 separate experimental replications, with the comprehensive quantitative values enumerated in Tables 5 and 6.

**Table 5.** Comprehensive evaluation of downstream tasks under the MAE framework

Annotation budget	1:1	1:5	1:10	1:50	1:100	1:500	1:1,000	1:5,000	1:10,000
MAE linear probing									
100%	0.92 ± 0.001	0.90 ± 0.000	0.89 ± 0.000	0.90 ± 0.001	0.89 ± 0.001	0.89 ± 0.001	0.89 ± 0.001	0.89 ± 0.001	0.89 ± 0.000
50%	0.92 ± 0.001	0.90 ± 0.000	0.89 ± 0.001	0.89 ± 0.001	0.88 ± 0.001	0.88 ± 0.001	0.88 ± 0.001	0.88 ± 0.002	0.88 ± 0.001
10%	0.89 ± 0.002	0.88 ± 0.003	0.87 ± 0.003	0.86 ± 0.003	0.85 ± 0.002	0.85 ± 0.003	0.86 ± 0.003	0.86 ± 0.003	0.86 ± 0.003
5%	0.88 ± 0.001	0.87 ± 0.002	0.86 ± 0.002	0.85 ± 0.001	0.84 ± 0.002	0.84 ± 0.002	0.84 ± 0.002	0.84 ± 0.002	0.84 ± 0.001
1%	0.87 ± 0.005	0.86 ± 0.007	0.85 ± 0.007	0.84 ± 0.004	0.83 ± 0.008	0.83 ± 0.004	0.83 ± 0.013	0.84 ± 0.009	0.84 ± 0.008
MAE fine-tuning									
100%	0.96 ± 0.002	0.96 ± 0.001	0.96 ± 0.001	0.96 ± 0.000	0.96 ± 0.002	0.96 ± 0.001	0.96 ± 0.002	0.96 ± 0.001	0.96 ± 0.000
50%	0.96 ± 0.001	0.95 ± 0.001	0.95 ± 0.002	0.95 ± 0.002	0.95 ± 0.002	0.95 ± 0.001	0.95 ± 0.001	0.95 ± 0.002	0.95 ± 0.001
10%	0.94 ± 0.001	0.93 ± 0.002	0.93 ± 0.002	0.93 ± 0.001	0.93 ± 0.002	0.93 ± 0.002	0.93 ± 0.002	0.93 ± 0.003	0.93 ± 0.002
5%	0.93 ± 0.003	0.92 ± 0.002	0.92 ± 0.004	0.92 ± 0.003	0.91 ± 0.002	0.92 ± 0.003	0.91 ± 0.004	0.91 ± 0.005	0.91 ± 0.004
1%	0.90 ± 0.010	0.89 ± 0.007	0.88 ± 0.007	0.87 ± 0.008	0.86 ± 0.009	0.87 ± 0.009	0.86 ± 0.011	0.86 ± 0.006	0.86 ± 0.010

MAE, masked autoencoder

**Table 6.** Comprehensive evaluation of downstream tasks under the SimCLR framework

Annotation budget	1:1	1:5	1:10	1:50	1:100	1:500	1:1,000	1:5,000	1:10,000
SimCLR linear probing									
100%	0.91 ± 0.001	0.91 ± 0.001	0.91 ± 0.001	0.90 ± 0.001	0.90 ± 0.001	0.90 ± 0.001	0.90 ± 0.001	0.91 ± 0.000	0.90 ± 0.001
50%	0.91 ± 0.001	0.90 ± 0.002	0.90 ± 0.002	0.89 ± 0.001	0.89 ± 0.001	0.89 ± 0.001	0.89 ± 0.001	0.90 ± 0.001	0.89 ± 0.001
10%	0.89 ± 0.006	0.87 ± 0.005	0.88 ± 0.003	0.87 ± 0.002	0.87 ± 0.002	0.87 ± 0.003	0.87 ± 0.003	0.87 ± 0.003	0.87 ± 0.002
5%	0.88 ± 0.004	0.86 ± 0.005	0.87 ± 0.004	0.86 ± 0.003	0.86 ± 0.004	0.86 ± 0.004	0.86 ± 0.003	0.86 ± 0.003	0.86 ± 0.003
1%	0.86 ± 0.006	0.84 ± 0.009	0.84 ± 0.007	0.83 ± 0.005	0.83 ± 0.004	0.83 ± 0.009	0.83 ± 0.008	0.83 ± 0.005	0.83 ± 0.005
SimCLR fine-tuning									
100%	0.96 ± 0.001	0.96 ± 0.001	0.96 ± 0.002	0.96 ± 0.001	0.95 ± 0.001	0.96 ± 0.001	0.96 ± 0.002	0.96 ± 0.002	0.96 ± 0.001
50%	0.95 ± 0.001	0.95 ± 0.002	0.95 ± 0.001	0.95 ± 0.002	0.95 ± 0.002	0.95 ± 0.003	0.95 ± 0.001	0.95 ± 0.001	0.95 ± 0.003
10%	0.93 ± 0.004	0.93 ± 0.003	0.93 ± 0.002	0.93 ± 0.003	0.92 ± 0.003	0.92 ± 0.002	0.93 ± 0.003	0.93 ± 0.003	0.93 ± 0.004
5%	0.93 ± 0.004	0.92 ± 0.003	0.92 ± 0.005	0.92 ± 0.004	0.91 ± 0.003	0.92 ± 0.004	0.93 ± 0.005	0.92 ± 0.005	0.92 ± 0.003
1%	0.89 ± 0.002	0.87 ± 0.004	0.87 ± 0.005	0.86 ± 0.005	0.85 ± 0.005	0.86 ± 0.004	0.89 ± 0.006	0.87 ± 0.005	0.87 ± 0.005

SimCLR, simple framework for contrastive learning

As the cervical cytology category imbalance increased from 1:1 to 1:10,000, the Acc of downstream tasks consistently and progressively declined across 5 distinct annotation budget groups from 1% to 100%. Additionally, during the transition from 1:1 to 1:100, there was a notable decline in Acc, with a maximum drop of approximately 4%. These findings indicate a growing impact of cervical cytology category imbalance on downstream tasks. It is particularly noteworthy that the impact of this imbalance becomes markedly more pronounced when constrained by a minimal annotation budget, identified here as 1%. Under such conditions, the extreme imbalance at a ratio of 1:10,000 leads to a substantial diminution in Acc for downstream tasks, in contrast to scenarios where a balanced distribution (1:1) is maintained. These findings emphasize a pivotal insight: the adverse effects of category imbalance in cervical cytology on downstream tasks’ performance are substantially exacerbated as the availability of annotation resources diminishes.

To complement the detailed Acc analysis, we additionally evaluated sensitivity (recall), specificity, precision, F1-score, and AUC, with results systematically reported in Tables S1 to S10. Across both MAE and SimCLR pre-training frameworks, these metrics exhibit trends especially under linear probing, where they align more closely with ACC. Specifically, increasing pre-training class imbalance leads to decreasing sensitivity and F1-score, indicating impaired positive-class detection, while specificity, precision, and AUC show modest declines.

Furthermore, transferring the pre-trained representations obtained during the SSRL stage to downstream tasks, these tasks can achieve results approximate to those using a substantial quantity of annotations (50%) with just a small fraction (5%) of annotations. This efficiently reduces the demand for annotations, facilitating a cost-effective annotation process.

## Conclusion and Discussion

The approach of employing SSRL without annotations and fine-tuning with a limited quantity of annotations has progressively emerged as a widely adopted paradigm in both the fields of computer vision and medical imaging. However, there has been limited work focusing on the impact of data imbalance on SSRL. In this work, we investigate the impact of extreme cervical cytology category imbalance on SSRL by transferring the pre-trained representations obtained from SSRL to downstream tasks with varying annotation budgets. Here, the impact of cervical cytology category imbalance on SSRL should be understood as an indirect effect: category imbalance changes the latent semantic composition of the unlabeled pre-training data, introduces sampling bias during pre-training, and thereby affects the learned representations and their downstream transferability.

Our analysis shows that Acc decreases notably in downstream tasks, up to 4%, as the data balance shifts from a 1:1 ratio to a highly imbalanced 1:100. This illustrates a clear relationship between the degree of cervical cytology category imbalance and its impact on SSRL while also indicating that this impact is manifested through pre-training-induced representation bias rather than a direct label-aware effect on the self-supervised objective. However, as the data balance reaches 1:100, a notable transition point emerges, where the performance stops declining and begins to stabilize. We attribute this phenomenon to a shift in the representation learning regime. At this stage, minority samples are too scarce to support stable feature learning for the minority class yet still numerous enough to disrupt the global feature space. Consequently, the model enters a regime in which minority signals are insufficient to form robust representations but still interfere with the overall feature space. This leads to the most pronounced performance drop around the 1:100 ratio. When the imbalance becomes even more extreme, the influence of minority samples further diminishes and the representation gradually shifts toward a more stable majority-dominated structure. As a result, the degradation no longer continues to increase.

From a methodological perspective, the results also suggest that different SSRL objectives may respond differently to category imbalance. Reconstruction-based methods such as MAE may still benefit from shared structural information in the images, whereas contrastive methods such as SimCLR may be more directly influenced by the frequency of dominant semantic patterns in the unlabeled pre-training pool. Although this work mainly focuses on the empirical impact of cervical cytology category imbalance on SSRL, the results indicate that such impact is closely related to the way that different self-supervised objectives form representations under skewed sampling conditions.

Beyond the impact of category imbalance, the model is particularly sensitive to variations in category distribution when dealing with limited annotations, especially under a 1% annotation budget. Furthermore, the downstream tasks demonstrate the potential to achieve accuracy comparable to those of scenarios with a high annotation budget (50%), even when utilizing a limited annotation budget (5%).

In our future research, we will focus on addressing the impact of cervical cytology category imbalance on SSRL. Currently, existing methods typically tackle data imbalance issues through data resampling. However, the distinctive nature of SSRL involves training on unlabeled data, making it essential to devise novel strategies to address this challenge. Since the categories of data are unknown a priori in SSRL, special consideration needs to be given to effectively handling imbalanced data categories and mitigating the representation bias induced during pre-training.

## Ethical Approval

This study was a retrospective analysis of archived cervical cytology slides. All slides were fully anonymized prior to analysis to ensure patient privacy protection. The use of archival materials was authorized and approved by the local ethical committee, and the study protocol adhered to all relevant institutional regulations and ethical guidelines.

## Data Availability

The data supporting the findings of this study are not publicly available because of privacy concerns.
