# Supplementary material for: The Impact of Cervical Cytology Category Imbalance on Self-Supervised Representation Learning
Source: Comput Struct Biotechnol J. 2026 Apr 24;35(1):0048. doi: 10.34133/csbj.0048 (PMC13106941; doi:10.34133/csbj.0048)
Supplement: Supplementary 1 — Tables S1 to S17 Fig. S1 [file csbj.0048.f1.zip › Supplementary.docx]

**Supplementary Table S1** Comprehensive evaluation of downstream tasks under the MAE Framework: Sensitivity

| Annotation Budget | MAE Linear Probing | | | | | | | | |
| --- | --- | --- | --- | --- | --- | --- | --- | --- | --- |
|  | 1:1 | 1:5 | 1:10 | 1:50 | 1:100 | 1:500 | 1:1K | 1:5K | 1:10K |
| 100% | 0.92±0.002 | 0.91±0.002 | 0.89±0.002 | 0.9±0.003 | 0.89±0.002 | 0.90±0.003 | 0.89±0.004 | 0.89±0.003 | 0.89±0.003 |
| 50% | 0.92±0.005 | 0.91±0.005 | 0.88±0.005 | 0.89±0.007 | 0.88±0.007 | 0.88±0.008 | 0.88±0.008 | 0.88±0.008 | 0.88±0.007 |
| 10% | 0.89±0.015 | 0.89±0.020 | 0.86±0.017 | 0.86±0.016 | 0.84±0.017 | 0.85±0.015 | 0.86±0.016 | 0.86±0.019 | 0.85±0.014 |
| 5% | 0.89±0.007 | 0.89±0.011 | 0.86±0.013 | 0.86±0.012 | 0.85±0.010 | 0.84±0.011 | 0.86±0.011 | 0.86±0.011 | 0.85±0.007 |
| 1% | 0.88±0.016 | 0.87±0.025 | 0.86±0.019 | 0.85±0.021 | 0.84±0.023 | 0.84±0.018 | 0.84±0.029 | 0.85±0.034 | 0.84±0.025 |
|  | MAE Fine-tune | | | | | | | | |
| 100% | 0.97±0.002 | 0.97±0.002 | 0.97±0.001 | 0.97±0.001 | 0.96±0.003 | 0.97±0.002 | 0.97±0.002 | 0.96±0.002 | 0.96±0.002 |
| 50% | 0.96±0.001 | 0.96±0.003 | 0.96±0.003 | 0.96±0.001 | 0.96±0.003 | 0.96±0.001 | 0.95±0.003 | 0.96±0.002 | 0.96±0.003 |
| 10% | 0.95±0.003 | 0.94±0.002 | 0.93±0.003 | 0.94±0.006 | 0.93±0.003 | 0.93±0.006 | 0.93±0.005 | 0.93±0.006 | 0.93±0.005 |
| 5% | 0.94±0.009 | 0.93±0.009 | 0.92±0.010 | 0.92±0.008 | 0.92±0.011 | 0.92±0.010 | 0.91±0.014 | 0.92±0.013 | 0.91±0.011 |
| 1% | 0.91±0.023 | 0.90±0.024 | 0.90±0.022 | 0.89±0.024 | 0.88±0.025 | 0.88±0.025 | 0.88±0.027 | 0.88±0.019 | 0.87±0.025 |

**Supplementary Table S2** Comprehensive evaluation of downstream tasks under the MAE Framework: Specificity

| Annotation Budget | MAE Linear Probing | | | | | | | | |
| --- | --- | --- | --- | --- | --- | --- | --- | --- | --- |
|  | 1:1 | 1:5 | 1:10 | 1:50 | 1:100 | 1:500 | 1:1K | 1:5K | 1:10K |
| 100% | 0.92±0.001 | 0.90±0.002 | 0.89±0.002 | 0.89±0.002 | 0.88±0.001 | 0.88±0.002 | 0.88±0.003 | 0.89±0.003 | 0.89±0.003 |
| 50% | 0.91±0.004 | 0.89±0.004 | 0.89±0.005 | 0.89±0.005 | 0.88±0.005 | 0.87±0.006 | 0.88±0.006 | 0.88±0.006 | 0.88±0.005 |
| 10% | 0.90±0.012 | 0.88±0.016 | 0.88±0.013 | 0.87±0.012 | 0.86±0.013 | 0.86±0.012 | 0.85±0.013 | 0.86±0.014 | 0.86±0.011 |
| 5% | 0.87±0.008 | 0.86±0.009 | 0.86±0.009 | 0.84±0.009 | 0.84±0.007 | 0.84±0.009 | 0.82±0.007 | 0.83±0.008 | 0.84±0.006 |
| 1% | 0.86±0.027 | 0.85±0.020 | 0.84±0.021 | 0.84±0.019 | 0.82±0.029 | 0.83±0.018 | 0.82±0.019 | 0.83±0.024 | 0.83±0.018 |
|  | MAE Fine-tune | | | | | | | | |
| 100% | 0.95±0.002 | 0.95±0.001 | 0.95±0.002 | 0.95±0.001 | 0.95±0.002 | 0.95±0.002 | 0.95±0.002 | 0.95±0.001 | 0.95±0.002 |
| 50% | 0.95±0.002 | 0.94±0.002 | 0.94±0.002 | 0.94±0.004 | 0.95±0.001 | 0.95±0.002 | 0.95±0.002 | 0.95±0.003 | 0.94±0.003 |
| 10% | 0.93±0.003 | 0.93±0.002 | 0.92±0.006 | 0.92±0.005 | 0.92±0.004 | 0.92±0.004 | 0.92±0.004 | 0.92±0.005 | 0.92±0.005 |
| 5% | 0.92±0.005 | 0.92±0.005 | 0.91±0.006 | 0.91±0.006 | 0.91±0.006 | 0.91±0.005 | 0.91±0.008 | 0.91±0.004 | 0.91±0.006 |
| 1% | 0.88±0.004 | 0.87±0.012 | 0.86±0.014 | 0.85±0.017 | 0.84±0.018 | 0.85±0.014 | 0.84±0.019 | 0.84±0.019 | 0.85±0.020 |

**Supplementary Table S3** Comprehensive evaluation of downstream tasks under the MAE Framework: F1-score

| Annotation Budget | MAE Linear Probing | | | | | | | | |
| --- | --- | --- | --- | --- | --- | --- | --- | --- | --- |
|  | 1:1 | 1:5 | 1:10 | 1:50 | 1:100 | 1:500 | 1:1K | 1:5K | 1:10K |
| 100% | 0.92±0.001 | 0.90±0.000 | 0.89±0.000 | 0.90±0.001 | 0.89±0.001 | 0.89±0.001 | 0.89±0.001 | 0.89±0.001 | 0.89±0.000 |
| 50% | 0.92±0.001 | 0.90±0.001 | 0.89±0.001 | 0.89±0.002 | 0.88±0.002 | 0.88±0.002 | 0.88±0.002 | 0.88±0.002 | 0.88±0.002 |
| 10% | 0.89±0.003 | 0.88±0.005 | 0.87±0.005 | 0.86±0.005 | 0.85±0.005 | 0.85±0.005 | 0.86±0.004 | 0.86±0.006 | 0.86±0.004 |
| 5% | 0.88±0.001 | 0.87±0.003 | 0.86±0.004 | 0.85±0.003 | 0.84±0.003 | 0.84±0.003 | 0.85±0.004 | 0.85±0.003 | 0.84±0.002 |
| 1% | 0.87±0.003 | 0.86±0.009 | 0.85±0.007 | 0.85±0.006 | 0.83±0.008 | 0.83±0.005 | 0.83±0.016 | 0.84±0.013 | 0.84±0.010 |
|  | MAE Fine-tune | | | | | | | | |
| 100% | 0.96±0.002 | 0.96±0.001 | 0.96±0.001 | 0.96±0.001 | 0.96±0.002 | 0.96±0.001 | 0.96±0.002 | 0.96±0.002 | 0.96±0.000 |
| 50% | 0.96±0.001 | 0.95±0.001 | 0.95±0.002 | 0.95±0.002 | 0.95±0.002 | 0.95±0.001 | 0.95±0.001 | 0.95±0.002 | 0.95±0.001 |
| 10% | 0.94±0.001 | 0.93±0.002 | 0.93±0.002 | 0.93±0.002 | 0.93±0.002 | 0.93±0.003 | 0.93±0.002 | 0.93±0.003 | 0.93±0.002 |
| 5% | 0.93±0.003 | 0.92±0.003 | 0.92±0.005 | 0.92±0.003 | 0.91±0.003 | 0.92±0.004 | 0.91±0.005 | 0.91±0.006 | 0.91±0.005 |
| 1% | 0.90±0.013 | 0.89±0.009 | 0.88±0.009 | 0.87±0.010 | 0.86±0.011 | 0.87±0.012 | 0.86±0.013 | 0.87±0.007 | 0.86±0.012 |

**Supplementary Table S4** Comprehensive evaluation of downstream tasks under the MAE Framework: AUC

| Annotation Budget | MAE Linear Probing | | | | | | | | |
| --- | --- | --- | --- | --- | --- | --- | --- | --- | --- |
|  | 1:1 | 1:5 | 1:10 | 1:50 | 1:100 | 1:500 | 1:1K | 1:5K | 1:10K |
| 100% | 0.97±0.000 | 0.96±0.000 | 0.96±0.000 | 0.96±0.000 | 0.95±0.000 | 0.96±0.000 | 0.95±0.000 | 0.95±0.000 | 0.95±0.000 |
| 50% | 0.97±0.000 | 0.96±0.000 | 0.95±0.000 | 0.95±0.000 | 0.95±0.000 | 0.95±0.000 | 0.95±0.000 | 0.95±0.000 | 0.95±0.000 |
| 10% | 0.96±0.001 | 0.95±0.001 | 0.94±0.002 | 0.94±0.003 | 0.92±0.002 | 0.93±0.003 | 0.93±0.002 | 0.93±0.002 | 0.93±0.003 |
| 5% | 0.95±0.001 | 0.94±0.001 | 0.93±0.000 | 0.93±0.002 | 0.91±0.002 | 0.92±0.002 | 0.92±0.003 | 0.92±0.002 | 0.92±0.002 |
| 1% | 0.94±0.005 | 0.94±0.004 | 0.93±0.007 | 0.92±0.005 | 0.90±0.008 | 0.91±0.004 | 0.91±0.010 | 0.92±0.008 | 0.91±0.006 |
|  | MAE Fine-tune | | | | | | | | |
| 100% | 0.99±0.000 | 0.99±0.000 | 0.99±0.000 | 0.99±0.000 | 0.99±0.001 | 0.99±0.000 | 0.99±0.000 | 0.99±0.000 | 0.99±0.000 |
| 50% | 0.99±0.001 | 0.98±0.001 | 0.98±0.001 | 0.98±0.001 | 0.98±0.001 | 0.98±0.001 | 0.98±0.000 | 0.98±0.001 | 0.98±0.001 |
| 10% | 0.98±0.001 | 0.97±0.001 | 0.97±0.000 | 0.97±0.001 | 0.97±0.001 | 0.97±0.001 | 0.97±0.001 | 0.97±0.001 | 0.97±0.001 |
| 5% | 0.98±0.001 | 0.97±0.001 | 0.97±0.003 | 0.97±0.002 | 0.97±0.002 | 0.97±0.002 | 0.97±0.003 | 0.97±0.003 | 0.97±0.002 |
| 1% | 0.96±0.005 | 0.95±0.004 | 0.95±0.006 | 0.94±0.004 | 0.94±0.006 | 0.94±0.006 | 0.94±0.007 | 0.94±0.005 | 0.94±0.007 |

**Supplementary Table S5** Comprehensive evaluation of downstream tasks under the MAE Framework: Precision

| Annotation Budget | MAE Linear Probing | | | | | | | | |
| --- | --- | --- | --- | --- | --- | --- | --- | --- | --- |
|  | 1:1 | 1:5 | 1:10 | 1:50 | 1:100 | 1:500 | 1:1K | 1:5K | 1:10K |
| 100% | 0.92±0.001 | 0.90±0.002 | 0.89±0.001 | 0.89±0.001 | 0.88±0.001 | 0.88±0.002 | 0.89±0.002 | 0.89±0.002 | 0.89±0.002 |
| 50% | 0.91±0.003 | 0.89±0.003 | 0.89±0.004 | 0.89±0.004 | 0.88±0.003 | 0.88±0.005 | 0.88±0.004 | 0.88±0.005 | 0.88±0.004 |
| 10% | 0.90±0.009 | 0.88±0.012 | 0.88±0.010 | 0.87±0.009 | 0.86±0.009 | 0.86±0.009 | 0.86±0.009 | 0.86±0.010 | 0.86±0.008 |
| 5% | 0.87±0.006 | 0.86±0.006 | 0.86±0.006 | 0.84±0.006 | 0.84±0.005 | 0.84±0.006 | 0.83±0.004 | 0.84±0.005 | 0.84±0.004 |
| 1% | 0.86±0.021 | 0.86±0.015 | 0.85±0.015 | 0.84±0.013 | 0.82±0.020 | 0.83±0.013 | 0.82±0.014 | 0.83±0.015 | 0.83±0.012 |
|  | MAE Fine-tune | | | | | | | | |
| 100% | 0.95±0.002 | 0.95±0.001 | 0.95±0.002 | 0.95±0.001 | 0.95±0.002 | 0.95±0.002 | 0.95±0.002 | 0.95±0.001 | 0.95±0.001 |
| 50% | 0.95±0.001 | 0.94±0.002 | 0.95±0.002 | 0.95±0.003 | 0.95±0.001 | 0.95±0.002 | 0.95±0.002 | 0.95±0.003 | 0.94±0.003 |
| 10% | 0.94±0.002 | 0.93±0.002 | 0.92±0.005 | 0.92±0.004 | 0.92±0.003 | 0.92±0.004 | 0.92±0.003 | 0.92±0.004 | 0.92±0.004 |
| 5% | 0.93±0.004 | 0.92±0.004 | 0.92±0.005 | 0.91±0.005 | 0.91±0.005 | 0.91±0.004 | 0.91±0.006 | 0.91±0.003 | 0.91±0.005 |
| 1% | 0.89±0.004 | 0.87±0.008 | 0.86±0.009 | 0.85±0.011 | 0.85±0.012 | 0.86±0.010 | 0.85±0.014 | 0.85±0.013 | 0.86±0.015 |

**Supplementary Table S6** Comprehensive evaluation of downstream tasks under the SimCLR Framework: Sensitivity

| Annotation Budget | SimCLR Linear Probing | | | | | | | | |
| --- | --- | --- | --- | --- | --- | --- | --- | --- | --- |
|  | 1:1 | 1:5 | 1:10 | 1:50 | 1:100 | 1:500 | 1:1K | 1:5K | 1:10K |
| 100% | 0.91±0.004 | 0.91±0.003 | 0.91±0.002 | 0.91±0.002 | 0.91±0.003 | 0.91±0.002 | 0.90±0.004 | 0.91±0.003 | 0.90±0.003 |
| 50% | 0.90±0.006 | 0.89±0.007 | 0.90±0.006 | 0.89±0.006 | 0.90±0.005 | 0.90±0.006 | 0.89±0.006 | 0.90±0.008 | 0.90±0.006 |
| 10% | 0.85±0.016 | 0.84±0.019 | 0.85±0.011 | 0.86±0.013 | 0.86±0.01 | 0.86±0.013 | 0.85±0.013 | 0.85±0.012 | 0.86±0.012 |
| 5% | 0.86±0.014 | 0.85±0.015 | 0.86±0.017 | 0.86±0.014 | 0.86±0.017 | 0.86±0.017 | 0.86±0.016 | 0.86±0.018 | 0.87±0.019 |
| 1% | 0.82±0.040 | 0.82±0.037 | 0.82±0.031 | 0.82±0.031 | 0.83±0.031 | 0.83±0.034 | 0.83±0.038 | 0.83±0.027 | 0.83±0.034 |
|  | SimCLR Fine-tune | | | | | | | | |
| 100% | 0.96±0.001 | 0.96±0.003 | 0.96±0.003 | 0.96±0.003 | 0.95±0.002 | 0.96±0.002 | 0.96±0.005 | 0.96±0.003 | 0.95±0.002 |
| 50% | 0.95±0.002 | 0.94±0.004 | 0.95±0.003 | 0.95±0.003 | 0.94±0.004 | 0.95±0.006 | 0.95±0.003 | 0.95±0.004 | 0.95±0.006 |
| 10% | 0.92±0.011 | 0.92±0.008 | 0.91±0.006 | 0.92±0.009 | 0.91±0.008 | 0.91±0.005 | 0.92±0.007 | 0.91±0.007 | 0.91±0.008 |
| 5% | 0.91±0.017 | 0.91±0.016 | 0.91±0.021 | 0.91±0.016 | 0.91±0.015 | 0.91±0.016 | 0.91±0.017 | 0.91±0.017 | 0.91±0.016 |
| 1% | 0.89±0.017 | 0.88±0.020 | 0.88±0.019 | 0.87±0.026 | 0.87±0.024 | 0.87±0.033 | 0.88±0.023 | 0.89±0.024 | 0.89±0.019 |

**Supplementary Table S7** Comprehensive evaluation of downstream tasks under the SimCLR Framework: Specificity

| Annotation Budget | SimCLR Linear Probing | | | | | | | | |
| --- | --- | --- | --- | --- | --- | --- | --- | --- | --- |
|  | 1:1 | 1:5 | 1:10 | 1:50 | 1:100 | 1:500 | 1:1K | 1:5K | 1:10K |
| 100% | 0.92±0.003 | 0.91±0.002 | 0.91±0.002 | 0.90±0.003 | 0.89±0.002 | 0.90±0.002 | 0.90±0.003 | 0.90±0.002 | 0.89±0.002 |
| 50% | 0.92±0.007 | 0.91±0.005 | 0.91±0.006 | 0.89±0.007 | 0.89±0.007 | 0.89±0.006 | 0.89±0.007 | 0.89±0.007 | 0.89±0.006 |
| 10% | 0.92±0.004 | 0.90±0.011 | 0.90±0.006 | 0.88±0.010 | 0.88±0.006 | 0.88±0.009 | 0.88±0.008 | 0.88±0.008 | 0.88±0.009 |
| 5% | 0.90±0.011 | 0.88±0.009 | 0.87±0.013 | 0.86±0.011 | 0.85±0.015 | 0.85±0.014 | 0.85±0.012 | 0.85±0.014 | 0.84±0.014 |
| 1% | 0.89±0.028 | 0.86±0.020 | 0.86±0.015 | 0.83±0.022 | 0.82±0.024 | 0.83±0.02 | 0.83±0.021 | 0.83±0.018 | 0.83±0.024 |
|  | SimCLR Fine-tune | | | | | | | | |
| 100% | 0.96±0.002 | 0.96±0.003 | 0.96±0.002 | 0.95±0.002 | 0.96±0.002 | 0.96±0.002 | 0.96±0.001 | 0.96±0.003 | 0.95±0.001 |
| 50% | 0.95±0.002 | 0.95±0.003 | 0.95±0.003 | 0.95±0.003 | 0.95±0.003 | 0.95±0.003 | 0.95±0.003 | 0.95±0.002 | 0.95±0.002 |
| 10% | 0.95±0.003 | 0.94±0.004 | 0.94±0.002 | 0.94±0.003 | 0.93±0.002 | 0.94±0.005 | 0.93±0.003 | 0.94±0.003 | 0.93±0.004 |
| 5% | 0.94±0.009 | 0.93±0.011 | 0.93±0.011 | 0.93±0.009 | 0.92±0.009 | 0.92±0.010 | 0.92±0.006 | 0.92±0.007 | 0.92±0.010 |
| 1% | 0.89±0.017 | 0.85±0.023 | 0.85±0.029 | 0.85±0.033 | 0.83±0.031 | 0.85±0.031 | 0.84±0.032 | 0.85±0.029 | 0.83±0.030 |

**Supplementary Table S8** Comprehensive evaluation of downstream tasks under the SimCLR Framework: F1-score

| Annotation Budget | SimCLR Linear Probing | | | | | | | | |
| --- | --- | --- | --- | --- | --- | --- | --- | --- | --- |
|  | 1:1 | 1:5 | 1:10 | 1:50 | 1:100 | 1:500 | 1:1K | 1:5K | 1:10K |
| 100% | 0.91±0.001 | 0.91±0.001 | 0.91±0.001 | 0.90±0.001 | 0.90±0.001 | 0.90±0.001 | 0.90±0.001 | 0.91±0.001 | 0.90±0.001 |
| 50% | 0.91±0.001 | 0.90±0.003 | 0.90±0.002 | 0.89±0.000 | 0.89±0.001 | 0.89±0.001 | 0.89±0.001 | 0.90±0.002 | 0.89±0.001 |
| 10% | 0.88±0.008 | 0.87±0.007 | 0.87±0.004 | 0.87±0.004 | 0.87±0.004 | 0.87±0.004 | 0.86±0.005 | 0.87±0.004 | 0.87±0.004 |
| 5% | 0.88±0.005 | 0.86±0.006 | 0.87±0.005 | 0.86±0.004 | 0.86±0.005 | 0.86±0.005 | 0.86±0.005 | 0.86±0.005 | 0.86±0.005 |
| 1% | 0.85±0.012 | 0.84±0.014 | 0.84±0.012 | 0.83±0.010 | 0.83±0.009 | 0.83±0.013 | 0.83±0.014 | 0.83±0.009 | 0.83±0.011 |
|  | SimCLR Fine-tune | | | | | | | | |
| 100% | 0.96±0.001 | 0.96±0.001 | 0.96±0.002 | 0.96±0.001 | 0.95±0.001 | 0.96±0.001 | 0.96±0.002 | 0.96±0.002 | 0.95±0.001 |
| 50% | 0.95±0.001 | 0.95±0.002 | 0.95±0.001 | 0.95±0.002 | 0.95±0.002 | 0.95±0.003 | 0.95±0.001 | 0.95±0.001 | 0.95±0.004 |
| 10% | 0.93±0.005 | 0.93±0.003 | 0.93±0.003 | 0.93±0.004 | 0.92±0.003 | 0.92±0.002 | 0.92±0.003 | 0.92±0.004 | 0.92±0.004 |
| 5% | 0.93±0.005 | 0.92±0.004 | 0.92±0.007 | 0.92±0.005 | 0.91±0.004 | 0.91±0.005 | 0.91±0.007 | 0.91±0.006 | 0.91±0.004 |
| 1% | 0.89±0.003 | 0.87±0.004 | 0.87±0.002 | 0.86±0.004 | 0.86±0.004 | 0.86±0.007 | 0.86±0.005 | 0.87±0.005 | 0.86±0.002 |

**Supplementary Table S9** Comprehensive evaluation of downstream tasks under the SimCLR Framework: AUC

| Annotation Budget | SimCLR Linear Probing | | | | | | | | |
| --- | --- | --- | --- | --- | --- | --- | --- | --- | --- |
|  | 1:1 | 1:5 | 1:10 | 1:50 | 1:100 | 1:500 | 1:1K | 1:5K | 1:10K |
| 100% | 0.97±0.000 | 0.97±0.000 | 0.97±0.000 | 0.96±0.000 | 0.96±0.000 | 0.96±0.000 | 0.96±0.000 | 0.96±0.000 | 0.96±0.000 |
| 50% | 0.97±0.000 | 0.96±0.000 | 0.96±0.000 | 0.96±0.000 | 0.96±0.000 | 0.96±0.000 | 0.96±0.000 | 0.96±0.000 | 0.96±0.001 |
| 10% | 0.96±0.002 | 0.95±0.001 | 0.95±0.001 | 0.94±0.002 | 0.94±0.002 | 0.94±0.002 | 0.94±0.002 | 0.94±0.001 | 0.94±0.001 |
| 5% | 0.95±0.002 | 0.94±0.002 | 0.94±0.002 | 0.93±0.003 | 0.93±0.003 | 0.93±0.003 | 0.93±0.002 | 0.93±0.003 | 0.93±0.003 |
| 1% | 0.93±0.002 | 0.92±0.005 | 0.92±0.003 | 0.91±0.004 | 0.90±0.003 | 0.91±0.007 | 0.91±0.005 | 0.91±0.003 | 0.91±0.003 |
|  | SimCLR Fine-tune | | | | | | | | |
| 100% | 0.99±0.000 | 0.99±0.000 | 0.99±0.000 | 0.99±0.000 | 0.99±0.000 | 0.99±0.001 | 0.99±0.000 | 0.99±0.001 | 0.99±0.000 |
| 50% | 0.99±0.000 | 0.98±0.001 | 0.98±0.001 | 0.98±0.001 | 0.98±0.001 | 0.98±0.001 | 0.98±0.001 | 0.98±0.001 | 0.98±0.001 |
| 10% | 0.98±0.001 | 0.98±0.001 | 0.98±0.001 | 0.98±0.001 | 0.97±0.000 | 0.97±0.001 | 0.97±0.001 | 0.97±0.001 | 0.97±0.001 |
| 5% | 0.98±0.001 | 0.97±0.001 | 0.97±0.001 | 0.97±0.001 | 0.97±0.001 | 0.97±0.002 | 0.97±0.002 | 0.97±0.002 | 0.97±0.001 |
| 1% | 0.95±0.002 | 0.94±0.004 | 0.94±0.002 | 0.94±0.003 | 0.93±0.003 | 0.94±0.005 | 0.94±0.004 | 0.94±0.002 | 0.93±0.004 |

**Supplementary Table S10** Comprehensive evaluation of downstream tasks under the SimCLR Framework: Precision

| Annotation Budget | SimCLR Linear Probing | | | | | | | | |
| --- | --- | --- | --- | --- | --- | --- | --- | --- | --- |
|  | 1:1 | 1:5 | 1:10 | 1:50 | 1:100 | 1:500 | 1:1K | 1:5K | 1:10K |
| 100% | 0.92±0.002 | 0.91±0.002 | 0.91±0.002 | 0.90±0.002 | 0.90±0.001 | 0.90±0.002 | 0.90±0.002 | 0.90±0.002 | 0.90±0.002 |
| 50% | 0.92±0.006 | 0.91±0.004 | 0.91±0.005 | 0.89±0.006 | 0.89±0.005 | 0.89±0.005 | 0.89±0.006 | 0.89±0.005 | 0.89±0.004 |
| 10% | 0.92±0.003 | 0.90±0.009 | 0.89±0.005 | 0.88±0.007 | 0.87±0.004 | 0.88±0.007 | 0.88±0.006 | 0.88±0.006 | 0.87±0.006 |
| 5% | 0.90±0.009 | 0.88±0.007 | 0.87±0.010 | 0.86±0.007 | 0.85±0.010 | 0.85±0.010 | 0.85±0.008 | 0.85±0.009 | 0.85±0.009 |
| 1% | 0.88±0.021 | 0.85±0.012 | 0.85±0.009 | 0.83±0.013 | 0.82±0.014 | 0.83±0.012 | 0.83±0.011 | 0.83±0.010 | 0.83±0.015 |
|  | SimCLR Fine-tune | | | | | | | | |
| 100% | 0.96±0.002 | 0.96±0.002 | 0.96±0.002 | 0.95±0.002 | 0.96±0.002 | 0.96±0.002 | 0.96±0.001 | 0.96±0.003 | 0.95±0.001 |
| 50% | 0.95±0.002 | 0.95±0.003 | 0.95±0.003 | 0.95±0.003 | 0.95±0.003 | 0.95±0.003 | 0.95±0.003 | 0.95±0.002 | 0.95±0.002 |
| 10% | 0.94±0.002 | 0.94±0.004 | 0.94±0.001 | 0.94±0.003 | 0.93±0.001 | 0.94±0.004 | 0.93±0.003 | 0.93±0.003 | 0.93±0.004 |
| 5% | 0.94±0.008 | 0.93±0.009 | 0.93±0.009 | 0.93±0.007 | 0.92±0.008 | 0.92±0.008 | 0.92±0.004 | 0.92±0.006 | 0.92±0.009 |
| 1% | 0.89±0.013 | 0.86±0.017 | 0.86±0.021 | 0.86±0.023 | 0.84±0.021 | 0.85±0.021 | 0.85±0.022 | 0.86±0.020 | 0.84±0.020 |

**Supplementary Table S11** MAE pretraining configuration

| parameter | value |
| --- | --- |
| Backbone | Vit-B/16 |
| Input size | 224×224 |
| Encoder | 12×transformer |
| Decoder | 12×transformer |
| Patch size for Vit | 16×16 |
| Mixed precision | AMP |
| Embedding dimension | 768 |
| MLP ratio | 4 |
| Batch size | 384 |
| Mask ratio | 0.5 |
| Optimizer | AdamW |
| Optimizer hyperparameters | *β*_1_=0.9, *β*_2_=0.95 |
| Total epochs | 200 |
| Weight decay | 0.05 |
| Learning rate | 2.25e-4 |
| Warmup epochs | 10 |
| Learning rate decay | Cosine annealing |
| Random seed | 0 |
| Data augmentation | Resize  RandomHorizontalFlip |

**Supplementary Table S12** SimCLR pretraining configuration

| parameter | value |
| --- | --- |
| Encoder | Resnet50 |
| Input size | 224×224 |
| Projection dimension | 128 |
| Temperature | 0.07 |
| Batch size | 512 |
| Optimizer | AdamW |
| Optimizer hyperparameters | *β*_1_=0.9, *β*_2_=0.95 |
| Total epochs | 200 |
| Weight decay | 1e-6 |
| Learning rate | 3e-3 |
| Warmup epochs | 10 |
| Learning rate decay | Cosine annealing |
| Random seed | 0 |
| Data augmentation | Resize  RandomHorizontalFlip  Color jitter  RandomGrayscale  GaussianBlur |

**Supplementary Table S13** MAE linear probing configuration

| parameter | value |
| --- | --- |
| Optimizer | SGD |
| Weight decay | 0 |
| Total epochs | 100 |
| Base learning rate | 1.5e-3 |
| Warmup epochs | 5 |
| Learning rate decay | Cosine annealing |
| Class token | True |
| Data augmentation | Resize  RandomHorizontalFlip |
| Pin memory | True |
| Number workers | 4 |

**Supplementary Table S14** MAE fine-tuning configuration

| parameter | value |
| --- | --- |
| Optimizer | AdamW |
| Optimizer hyperparameters | *β*_1_=0.9, *β*_2_=0.95 |
| Total epochs | 50 |
| Base learning rate | 5e-4 |
| Warmup epochs | 5 |
| Learning rate decay | Cosine annealing |
| Data augmentation | Resize  RandomHorizontalFlip |
| Pin memory | True |
| Number workers | 4 |

**Supplementary Table S15** SimCLR linear probing configuration

| parameter | value |
| --- | --- |
| Optimizer | SGD |
| Weight decay | 0 |
| Total epochs | 100 |
| Base learning rate | 1.5e-3 |
| Warmup epochs | 5 |
| Learning rate decay | Cosine annealing |
| Data augmentation | Resize  RandomHorizontalFlip |
| Pin memory | True |
| Number workers | 4 |

**Supplementary Table S16** SimCLR fine-tuning configuration

| parameter | value |
| --- | --- |
| Optimizer | AdamW |
| Optimizer hyperparameters | *β*_1_=0.9, *β*_2_=0.95 |
| Total epochs | 50 |
| Base learning rate | 5e-4 |
| Warmup epochs | 5 |
| Learning rate decay | Cosine annealing |
| Data augmentation | Resize  RandomHorizontalFlip |
| Pin memory | True |
| Number workers | 4 |

**Supplementary Table S17** Linear SVM classification performance on original 768-dimensional embeddings under different pre-training strategies (ImageNet vs. MAE) and annotation budgets (1% vs. 100%). Metrics include Accuracy, Specificity, Sensitivity, Precision, F1-score, and AUC. Results indicate that MAE pre-training consistently improves linear separability, especially under limited annotations.

| criterion | ImageNet | | | MAE | | |
| --- | --- | --- | --- | --- | --- | --- |
|  | pretrain | FN_1% | FN_100% | pretrain | FN_1% | FN_100% |
| Accuracy | 0.82±0.012 | 0.86±0.011 | 0.95±0.012 | 0.89±0.019 | 0.90±0.017 | 0.95±0.008 |
| Specificity | 0.81±0.019 | 0.86±0.017 | 0.94±0.026 | 0.89±0.030 | 0.90±0.025 | 0.95±0.022 |
| Sensitivity | 0.82±0.033 | 0.86±0.015 | 0.95±0.020 | 0.89±0.023 | 0.91±0.011 | 0.94±0.018 |
| Precision | 0.82±0.012 | 0.86±0.015 | 0.94±0.023 | 0.89±0.028 | 0.90±0.024 | 0.95±0.020 |
| F1 score | 0.82±0.016 | 0.86±0.011 | 0.95±0.011 | 0.89±0.018 | 0.90±0.016 | 0.95±0.008 |
| AUC | 0.90±0.008 | 0.92±0.010 | 0.98±0.007 | 0.95±0.007 | 0.96±0.009 | 0.97±0.008 |


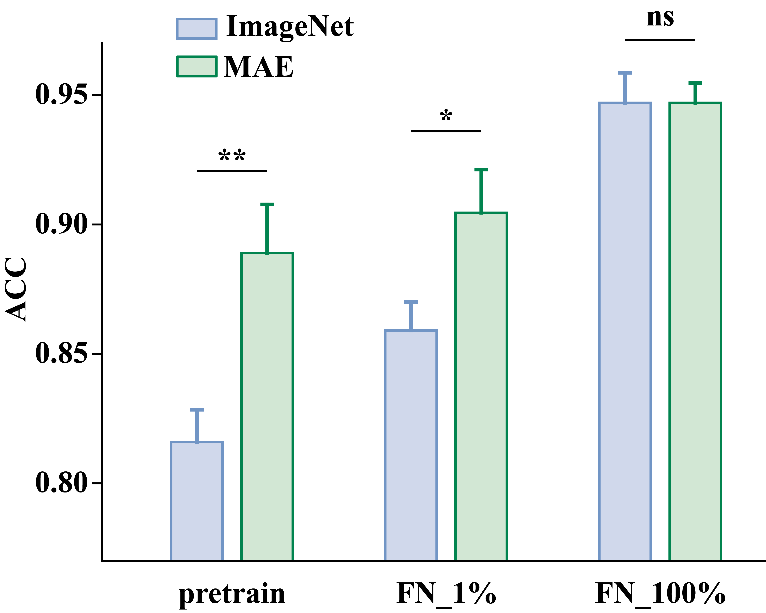


**Supplementary Table 1** Linear SVM classification accuracy (ACC) on original embeddings under varying pre-training strategies and annotation budgets. Error bars indicate standard deviation. MAE pre-training yields higher ACC than ImageNet pre-training, consistent with qualitative t-SNE observations, demonstrating improved linear separability of learned feature representations.
