# Supplementary figures and images for: The Impact of Cervical Cytology Category Imbalance on Self-Supervised Representation Learning
Source: Comput Struct Biotechnol J. 2026 Apr 24;35(1):0048. doi: 10.34133/csbj.0048 (PMC13106941; doi:10.34133/csbj.0048)

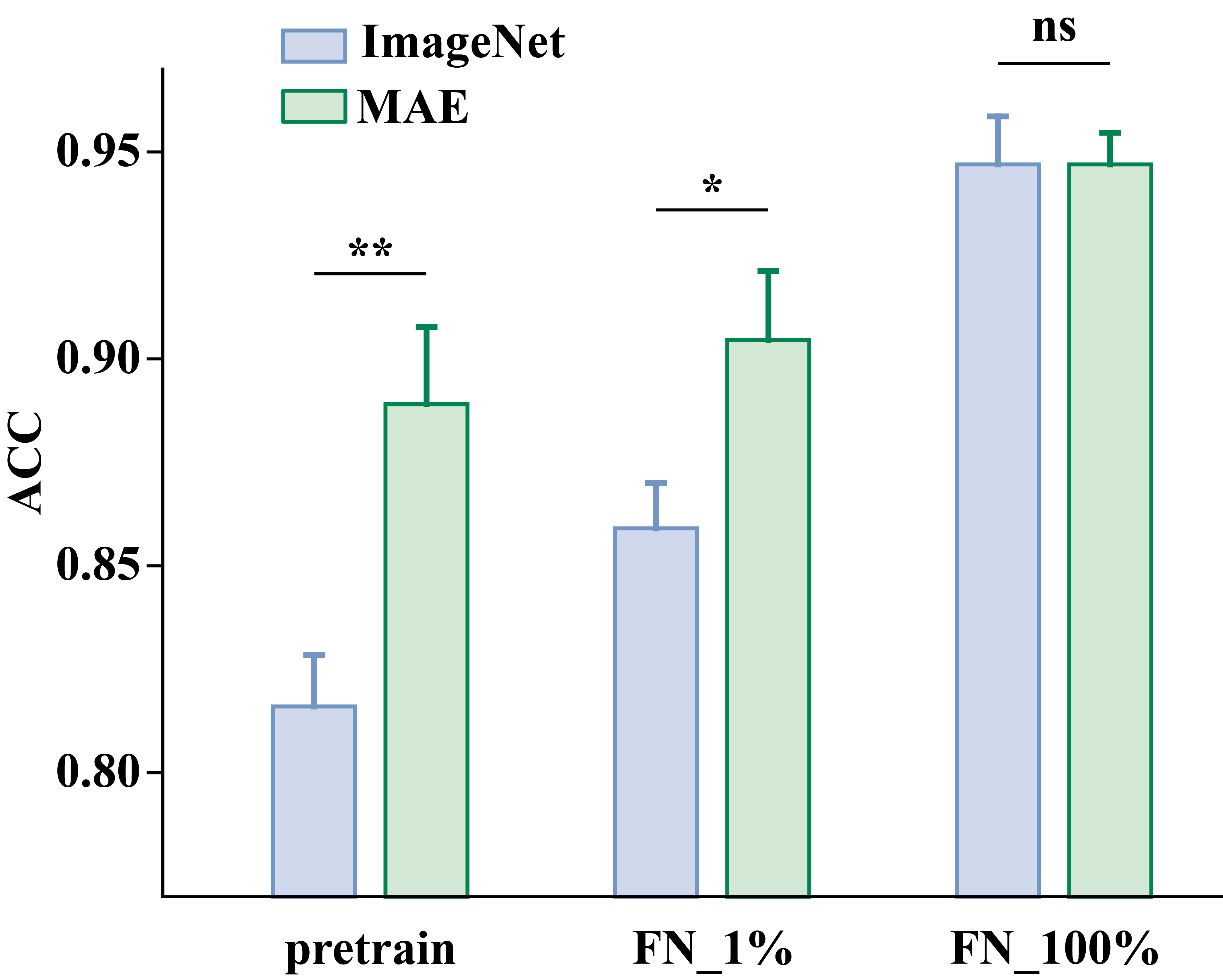

Supplement: Supplementary 1 — Tables S1 to S17 Fig. S1 [file csbj.0048.f1.zip › Supplementary Figure 1 .png]
